# Human Bone Marrow Mesenchymal Stem/Stromal Cells Preserve Their Immunomodulatory and Chemotactic Properties When Expanded in a Human Plasma Derived Xeno-Free Medium

**DOI:** 10.1155/2017/2185351

**Published:** 2017-05-14

**Authors:** A. Blázquez-Prunera, C. R. Almeida, M. A. Barbosa

**Affiliations:** ^1^Instituto de Investigação e Inovação Em Saúde, Universidade do Porto, Porto, Portugal; ^2^Instituto de Engenharia Biomédica, Universidade do Porto, Porto, Portugal; ^3^Faculdade de Engenharia, Universidade do Porto, Porto, Portugal; ^4^Instituto de Ciências Biomédicas Abel Salazar, Universidade do Porto, Porto, Portugal; ^5^Department of Medical Sciences and Institute for Biomedicine (iBiMED), University of Aveiro, 3810-193 Aveiro, Portugal

## Abstract

Due to their immunomodulatory and chemotactic properties, hMSC are being explored to treat immune-related diseases. For their use in human therapies, it is necessary to culture hMSC in xeno-free conditions. In this study, the impact that a xeno-free medium based on a human plasma derivate has on these properties was analysed. Bone marrow-derived hMSC preserved their immunosuppressive and immunostimulatory properties, as observed with in vitro assays with hMSC cocultured with mixed leukocyte reactions or with mitogen-stimulated leukocytes. Moreover, hMSC expanded in xeno-free medium were recruited by macrophages in both migration and invasion assays, which indicates that the cells maintained their chemotactic properties. These data suggest that xeno-free expanded hMSC preserved their immunomodulatory and chemotactic properties, indicating that the described xeno-free medium composition is a potential candidate to culture and expand hMSC for human cell therapies.

## 1. Introduction

Human mesenchymal stem/stromal cells (hMSC) are a promising tool in regenerative medicine and for treatment of immune-mediated diseases [1]. There are currently more than 600 clinical trials evaluating the use of hMSC for different therapies (search of term “Mesenchymal Stem/Stromal Cell”) [2]. Some of these therapies are related with the differentiation capacity of hMSC to promote bone repair or to regenerate cartilage [3, 4], while others are related with the immunomodulatory properties, the capacity to secrete trophic factors, to promote vascularization and to inhibit cell death, such as in graft-versus-host-disease (GvHd), kidney transplant rejection, ischemic cardiomyopathy, and progressive multiple sclerosis amongst other applications [5–8].

hMSC exert immunomodulatory effects on cells of both the adaptive and innate immune systems, in a way that depends on the source of hMSC, the number of passages in culture, the specific niche where hMSC are [9] the type of culture (in suspension or attached) [10, 11], and the confluence of the culture [12, 13]. The balance between the stimuli received by hMSC determines the acquisition of an immunosuppressive or an immunostimulatory behaviour [14]. The mechanism through which hMSC exert their immunomodulatory action is not fully understood but it is known that hMSC must be primed to produce immunosuppressive mediators. Proinflammatory factors secreted by immune cells, such as tumor necrosis factor alpha (TNF-*α*) and interferon gamma (IFN-*γ*), can prime hMSC, inducing synthesis of prostaglandin E2 (PGE2) and activating the production of indoleamine 2,3-dioxygenase (IDO). These two molecules play an important role in hMSC immunosuppressive properties [15–18].

Many of the currently explored clinical strategies take advantage of the hMSC capacity of homing to an injury or inflammation site. And indeed, recruitment of MSC to a bone injury has been correlated with its repair [19, 20]. Some studies have tackled which mediators, including cytokines, stimulate and regulate this recruitment [21, 22]. And it has become clear that inflammatory mediators lead to increased MSC migration, by directly recruiting the cells, or by stimulating production of matrix-degrading enzymes [23] or even promoting expression of homing-related molecules by the MSC [24]. Thus, immune cells that produce these mediators, such as NK cells, macrophages, and T cells, can attract MSC [25–27, 22]. Macrophages are one of the most abundant cell types in an injury area and are particularly strong recruiters of hMSC, through their secretion of soluble mediators [22, 28].

An important point to consider regarding the application of hMSC in clinical therapies is their expansion under xeno-free conditions. hMSC are usually cultured in media supplemented with foetal bovine serum (FBS), which is inherently risky, as cells are exposed to bovine immunogenic proteins. Furthermore, FBS presents high variability between lots and its availability is limited [29]. Different human-derived supplements, such as human serum or human platelet lysate, are thus being developed to substitute the use of FBS in the culture of hMSC [30–35]. Supplementation with human autologous serum is an interesting approach but that nevertheless presents some disadvantages such as donor variability and its availability [36, 37]. The use of platelet lysate has led to promising results on hMSC expansion [38], but, again with variability depending on the donor and if the supplement is made from one single or a low number of donations [39]. Moreover, the impact of platelet lysate on the immunomodulatory properties of hMSC is controversial, with some studies suggesting that it dampens the immunossupressive capabilities of the cells [40, 41], besides affecting expression of adipogenic and osteogenic markers [42, 43]. A promising chemically defined medium was approved by FDA (StemPro MSC SFM, Invitrogen) to isolate and expand hMSC. Different studies were done to show its suitability to culture hMSC [36, 44], but due to the lack of standardization of the protocols, the results obtained are difficult to compare. Some studies reported a reduction on the potential for hMSC differentiation and on expression of some proteins, when compared with FBS-supplemented medium [36]. Bobis-Wozowicz and colleagues made a comparative study of different commercially available xeno-free media for hMSC expansion [45]. It was found that each medium had a different impact on hMSC properties, and thus, media for hMSC expansion should be chosen depending on the characteristics needed for a specific therapy [45].

With the objective of expanding hMSC for human cell therapies, the pharmaceutical company Grifols has developed a supplement for cell culture (SCC) [46]. SCC is derived from a human plasma specifically collected for the production of plasma-derived therapeutic products, following GMP rules. Plasma pools from over 1000 healthy donors undergo a cold ethanol industrial fractionation to obtain different drugs, including SCC [47]. Previous reports have shown that medium supplemented with SCC can be successfully used to culture hMSC, iPSC, ES, and other mammalian cell lines [48–50]. It has been shown that hMSC cultured with SCC remain undifferentiated in culture and preserve their adipogenic, osteogenic, and chondrogenic differentiation potential [50], but the impact on immunomodulation and chemotaxis remains unknown.

hMSC expanded in SCC-containing medium maintain their adherence to plastic, phenotype, and multipotentiality [50]. Here, we went further to investigate the influence that SCC has on two properties of hMSC that are important for cell therapy. First of all, we investigated the immunomodulatory properties of xeno-free expanded hMSC. hMSC can prime or suppress different cells of the immune system to modulate an immune response and facilitate tissue recovery [51, 52]. To investigate hMSC immunomodulation, proliferation of resting or stimulated leukocytes was quantified when cultured with hMSC. Secondly, the chemotactic properties of xeno-free expanded hMSC were also addressed. If hMSC are used for cell therapy, it is important that the hMSC injected go to the site of injury; thus, hMSC need to be recruited by cells and cytokines present in the inflamed tissue. Here, hMSC motility and chemotaxis promoted by macrophages were analysed by time-lapse microscopy and using transwell systems.

## 2. Material and Methods

### 2.1. Xeno-Free Medium Preparation

The xeno-free medium used for this study was similar to the one used by Díez et al. [50], but in this present study, platelet lysate and basic fibroblast growth factor were not added. Thus, the final composition used was DMEM (Life Technologies) supplemented with 15% supplement for cell culture (SCC, Grifols), 10 mg/L insulin (Life Technologies), 6.7 *μ*g/L sodium selenite (Sigma-Aldrich), 2 *μ*L/L ethanolamine (Sigma-Aldrich) [ISE], and 10 g/L penicillin/streptomycin (Gibco)—“XF-Medium” [50]. SCC is supplied in a freeze-dried format, which was reconstituted in 50 mL DMEM. After reconstitution, medium was filtered with 0.4 *μ*m and 0.2 *μ*m filters. Control medium was the commonly used to expand hMSC: DMEM supplemented with 10% hMSC-qualified FBS (Hyclone) and 1% penicillin/streptomycin (Gibco)—“FBS-Medium”.

### 2.2. Origin and Culture of hMSC

hMSC were isolated from human bone marrow by density gradient centrifugation and selection of adherent cells as in Almeida et al. [25]. Bone marrow collection was approved by “Comissão de Ética do Centro Hospitalar de S. João”. After written consent, bone marrow was obtained from discarded bone tissues of 3 different patients (females, 40, 52, and 56 years old) undergoing total hip arthroplasty at the Hospital of São João (Porto), whom did not present known inflammatory diseases. After isolation and expansion in FBS-Medium for two passages, cells were resuspended in media with 10% DMSO and placed in a Mr. Frosty freezing container which was placed at −80°C for approximately 24 hr, before transferring the cells to liquid nitrogen. Before performing any assay and due to practical reasons, cells were moved to a −80°C freezer, where they stayed for a maximum period of one month.

To perform the following studies, frozen aliquots of hMSC were thawed and firstly cultured in FBS-Medium for one passage: hMSC were grown at 37°C and 5% CO_2_ until reaching 80% confluence, when they were detached by treatment with xeno-free trypsin (TrypLE™ Express, Life Technologies) and counted using Trypan Blue. hMSC were then seeded in T-150 flasks at 3000 cells/cm^2^ in either FBS-Medium, as control, or XF-Medium and expanded between one and four passages before any assay was performed. Assays were performed with hMSC in passages ranging from 5 to 10.

### 2.3. Cell Growth Kinetics-Population Doubling Time

To calculate population doubling time (PDT), 5 × 10^4^ hMSC were seeded in T-25 flasks and passaged every 4 days for two passages in XF-Medium or FBS-Medium. At each passage, hMSC were detached using xeno-free trypsin and counted by Trypan Blue exclusion. Briefly, cells were washed with PBS and incubated with 1 mL trypsin for 2 minutes at 37°C. Then, 1 mL of medium was added to stop the reaction and cells were centrifuged. PDT was determined by the formula PDT = 1/[3.32 (log NH − log N1)/(t2 − t1)], where N1 is the inoculated cell number, NH is the cell number at harvest, t1 is the time at seeding, and t2 is the time at harvesting [53].

### 2.4. Immunomodulatory Properties

#### 2.4.1. Immunoregulation Assay—MLR

An immunosuppression assay was implemented to study hMSC capacity of immunomodulation after expansion in XF-Medium. Each mixed leukocyte reaction (MLR) was performed with peripheral blood mononuclear cells (PBMCs). PBMCs from one randomly selected donor were used as responder cells, whose proliferation was evaluated. As stimulator cells, we used PBMCs from 5 different donors to increase the likelihood to induce an immune response by the responder cells (Supplementary Figure 1 available online at https://doi.org/10.1155/2017/2185351). The stimulator cells were treated with a mitosis inhibitor to avoid their proliferation and thus interference in the analysis of proliferating responder cells [54–56]. PBMCs were isolated from buffy coat residues of unrelated healthy volunteers (following the approval and recommendations of the Ethics Committee for Health from Centro Hospitalar S. João (Porto—References 259/11 and 260/11)) by density-gradient centrifugation (Lymphoprep™; Axis-Shield). Briefly, buffy coats were diluted in PBS and overlaid carefully on Lymphoprep at a 2 : 1 ratio. Tubes were then centrifuged during 30 min at 800*g* with no break and lowest acceleration at room temperature. The PBMC layer was collected and washed 3 times by adding cold PBS up to 40 mL and centrifuging at 300*g* during 10 min. Finally, cells were resuspended in 10 mL PBS.

To obtain a stock of stimulator cells, PBMCs from 5 different donors were mixed, with the same quantity of cells from each donor, at a final concentration of 5 × 10^8^ cells/mL in PBS and 10% dimethyl sulfoxide (DMSO, Sigma-Aldrich). Aliquots were frozen and stored at −80°C until required. For the MLR, proliferation of stimulator cells was inhibited with mitomycin C (Sigma-Aldrich). First, the stimulator stock vial was thawed and washed and 5 × 10^7^ cells were resuspended in 1 mL PBS. Mitomycin C was then added at a final concentration of 50 *μ*g/mL and incubated for 20 min at 37°C. After the incubation time, an excess of RPMI supplemented with 5% FBS was added. The suspension was washed in this medium 3 times to ensure that there was no remaining mitomycin C in the sample. Finally, cells were resuspended in serum-free RPMI (GIBCO, RPMI-1640 Medium (1×) + GlutaMax; Life Technologies). Freshly isolated PBMCs from another donor were used as responder cells, which were labelled with CFSE (CellTrace™ CFSE; Life Technologies) by incubating 10^7^ cells/mL in PBS with 0.5 *μ*M CFSE for 15 min at 37°C (concentration optimized in our laboratory). After washing twice with PBS supplemented with 20% FBS, cells were resuspended in 1 mL RPMI.

To study hMSC immunomodulatory properties, 5 × 10^4^ hMSC were seeded in a 24-well flat-bottom tissue culture plate in XF-Medium or FBS-Medium and cultured overnight. Then, 5 × 10^5^ CFSE-labelled PBMCs (responder cells, R) and 5 × 10^5^ PBMCs (stimulator cells, S) were added to hMSC-containing wells, or to new wells, in RPMI-1670 with 2 mM L-glutamine (Life Technologies) supplemented with 10% FBS or 15% SCC (depending on the media used for hMSC expansion). After 6 days of culture at 37°C and 5% CO_2_, supernatants were kept at −80°C for future quantification of cytokines. Cells were harvested with trypsin and fixed using a 4% solution of paraformaldehyde (PFA, Sigma-Aldrich). Fixed samples were filtered using a 100 *μ*m pore size nylon membrane and analysed by flow cytometry in a FACSCalibur instrument. Data obtained were analysed using FLowJo software.

#### 2.4.2. Immunoregulation Assay—Mitogenic Stimulation (PHA)

The immunomodulatory properties of hMSC on mitogen-stimulated responder cells were also studied. The protocol used was similar to the MLR: hMSC were seeded and incubated overnight and the day after, responder cells were added. When indicated, 5 *μ*g/mL phytohemagglutinin (PHA, Sigma-Aldrich) was added to the medium. In this way, the hMSC immunosuppressive properties were studied towards mitogen-stimulated responder cells, whereas the immunostimulatory properties were studied upon coculture with nonstimulated responder cells.

#### 2.4.3. Cytokine Quantification

The medium of the different conditions was collected at the end of the assay and centrifuged at 400*g* for 10 min. Supernatants were collected and stored at −80°C until ELISA analysis. TNF-*α* was measured with a human TNF-alpha ELISA Kit (RayBio). Immunoassays were performed according to manufacturer's instructions. Absorbance of ELISA plates was read on a microplate reader (Sunrise™ Tecan).

### 2.5. Monocyte Isolation and Differentiation to Macrophages

Monocyte-enriched populations were obtained from human buffy coats from 4 different healthy donors (kindly provided from Centro Hospitalar S. João), by negative selection using a Tetrameric Antibody Complexes kit (RosetteSep, StemCell Technologies) as in Almeida et al. [25]. Macrophages were obtained by allowing in vitro differentiation of the isolated monocytes: 2 × 10^5^ monocytes/well were plated in 24-well companion plates (Falcon) and cultured at 37°C, 5% CO_2_ during 10 days in RPMI medium supplemented with 10% FBS. The purity of macrophages was determined examining cell morphology under the microscope and by analysis of CD14 and human leukocyte antigen- (HLA-) DR expression by flow cytometry, which was found to be 70–85%.

### 2.6. Migration and Invasion Assay

Recruitment assays were performed in 24-well plates using inserts with a membrane with 8 *μ*m pores (BD Biosciences). Membranes were incubated before the assay with 100 *μ*L of bovine gelatine (0.1% in PBS) for 1 h at 37°C and washed with PBS. Invasion assays were performed with an insert with the same characteristics, but Matrigel coated (Corning® BioCoat™ Matrigel™). These membranes were incubated with DMEM for 1 h at 37°C. After the incubation time, the lower compartments of the chambers were filled with 750 *μ*L DMEM, to evaluate basal motility, DMEM with 30% FBS, which contains a mix of soluble stimuli that recruit different cells types, as a positive control, or DMEM with macrophages (approximately 2 × 10^5^ cells/well), as an inflammatory stimuli. Macrophages were not harvested with the recruitment assay being performed in the same plate where monocytes were platted and allowed to differentiate. Then, 4 × 10^4^ hMSC previously expanded in XF-Medium and FBS-Medium were seeded into the upper compartment in 500 *μ*L DMEM, obtaining a 1 : 5 hMSC : macrophage ratio. hMSC from 3 different donors were used.

Chambers were incubated at 37 °C, 5% CO_2_ for 7 hours, in the case of migration assays, and 24 hours for invasion assays. After incubation, membranes were washed with PBS and cells were fixed with 4% PFA for 15 min at RT, followed by a washing step with PBS. Inserts were kept in PBS at 4°C until analysis. For analysis, cells on the top part of the membrane were removed with a cotton swab, and the membrane was cut and mounted on a slide with 4 *μ*L Vectashield-mounting medium with DAPI (Vector Laboratories). Cells that migrated were counted on an inverted fluorescence microscope at ×200 fields of view. An average of the number of cells in ×10 fields of view was calculated for each membrane. Chemotactic indexes were calculated by dividing the average number of hMSC that crossed the membrane in the experimental condition by the average number of hMSC that crossed the membrane in the negative control.

### 2.7. Time-Lapse Assay

To study the influence that SCC had on hMSC motility, 5000 hMSC from three different donors expanded in XF-Medium or FBS-Medium were seeded in a 24-well plate in serum-free DMEM medium (without SCC, FBS, or ISE). After overnight incubation, image acquisition was performed every 10 minutes during 12 hours at 37°C and 5% CO_2_ using the IN Cell Analyser 2000 (GE Healthcare). Images from four randomized positions per well were obtained in brightfield with a 10x objective. The percentage of motile cells was calculated by dividing the number of motile cells by the total number of cells that appeared in each video. Cell velocity of three randomly selected cells in each video was measured using Fiji program.

### 2.8. Morphological Evaluation

Morphometric analysis of hMSC in the different media was performed from 50x microscope images. Cells in contact with the border were discarded. The area and “circularity” of each cell were determined with ImageJ software. Circularity is calculated by ImageJ software using the formula 4pi(area/perimeter^2^). A circularity value of 1 indicates a perfect circle and as it approaches 0, it indicates an increasingly elongated polygon [57].

### 2.9. Statistical Analysis

Statistical analysis was performed using GaphPad Prism software, v5.01. Normal distribution of populations was verified with D'Agostino-Pearson omnibus test. The comparison of two populations was performed using the parametric paired or unpaired *t*-test or Mann-Whitney test (unpaired test). The comparison of three or more samples was done using the nonparametric Kruskal-Wallis test, followed by Dunn's multiple comparison test. Differences between samples were considered statistically significant when *p* values were <0.05 (∗), <0.01 (∗∗), and <0.001 (∗∗∗).

## 3. Results

### 3.1. Xeno-Free hMSC Preserve Their Immunomodulatory Properties

In order to determine the impact of XF-medium on MSC immunomodulatory properties, cells were expanded in medium-containing SCC, similar to the one used by Díez et al. [50]. However, here, basic fibroblast growth factor and platelet lysate were not added as the later might impact on MSC immunomodulation. The population doubling time (PDT) of cells expanded in this medium was calculated with hMSC from two different donors. The average PDT was 2.7 days for cells in FBS-Medium and 7.4 days for cells in XF-Medium, indicating that hMSC can be expanded in the xeno-free conditions defined here, even though taking 4 to 5 days more to duplicate their population when compared to the control.

**Figure 1:**
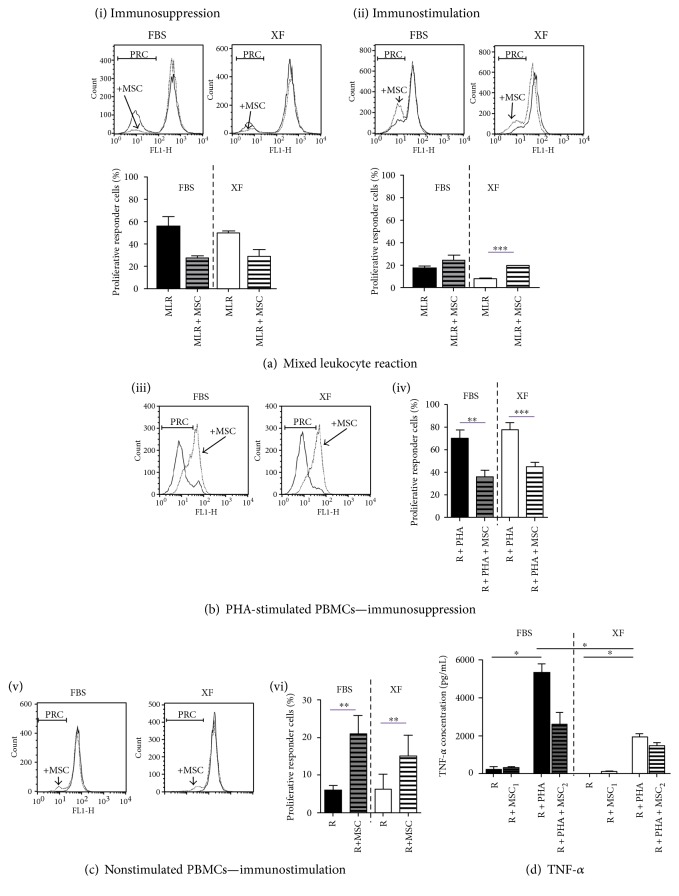
Immunomodulation by xeno-free MSC. (a) Analysis of the percentage of proliferative responder cells (PRC) present in a mixed leukocyte reaction (MLR) in the absence (continuous line) or in the presence of MSC (discontinuous line, indicated by “+MSC”). Representative comparative histograms (top) and bar graphs (bottom) of immunosuppression (i) and immunostimulation (ii) (*n* = 4–18, with 3 different hMSC donors; paired *t*-test). (b) Immunosuppression—proliferation of R cells was stimulated with PHA. (iii) Histograms show examples of hMSC immunosuppression (expanded in FBS- or XF-Medium). (iv) Summary of the collected data—the percentage of proliferative R is indicated (*n* = 8 − 9, with 3 different hMSC donors; paired *t*-test). (c) Immunostimulation—proliferation rate of resting PBMCs compared with the one obtained when coculturing the same cells with hMSC. (v) Histograms of these two conditions with hMSC expanded in FBS-Medium or XF-Medium. (vi) Summary of the collected data—the percentage of proliferative R is indicated (*n* = 8, with 3 different hMSC donors, paired *t*-test). (d) TNF-*α* production in different cultures. Supernatants of resting PBMCs (responder cells, R) or PBMCS stimulated with 5 *μ*g/mL PHA (R + PHA) in coculture or not with hMSC were collected and analysed by ELISA. Assays were done in FBS-containing medium when hMSC were expanded in FBS-Medium or in SCC-containing medium for hMSC expanded in XF-Medium (*MSC1: hMSC with immunostimulatory properties; MSC2: hMSC with immunosuppressive properties*) (*n* = 4, Kruskal-Wallis test followed by Dunn's multiple comparison test).

The immunomodulatory capability of hMSC was then analysed by quantifying inhibition of proliferation of alloreactive T-lymphocytes in mixed leukocyte reactions (MLR; Supplementary Figure 1). The results obtained were diverse, but two distinct patterns could be observed (Figure 1(a)), corresponding to the two MSC types defined by Waterman et al.: MSC1, for immunostimulatory MSC, and MSC2, for immunosuppressive MSC [58]. Generally, even though differences were not statistically significant, when the percentage of proliferative responder cells was higher than 30% in the control MLR (without hMSC), adding hMSC resulted in suppression of this proliferation (43.71 ± 21.23% reduction of proliferative cell number) (Figure 1(a), i). On the other hand, when the percentage of proliferative cells was lower than 30% in the control MLR, hMSC did not affect T cell proliferation or acted as immunostimulatory agents and led to an increase in the percentage of proliferative cells (208.2 ± 451.2%) (Figure 1(a), ii). This trend was observed both for hMSC expanded in XF-Medium and for hMSC expanded in FBS-Medium (Figure 1(a)).

As the MLR did not always lead to high proliferation of responder cells, and in order to obtain more reproducible data, the capacity of hMSC to suppress proliferation was studied against mitogen-stimulated PBMCs, as performed elsewhere [16, 59]. As shown in Figure 1(b), hMSC cultured in xeno-free conditions were able to inhibit proliferation of mitogen-stimulated PBMCs. When cells were stimulated with 5 *μ*g/mL PHA, the mean percentage of proliferative cells was 70 ± 21% (medium-containing FBS) and 76 ± 16% (medium-containing SCC) and the presence of MSC led to a mean reduction of 43 ± 13% for cells expanded in XF-Medium and 46 ± 23% when using FBS-Medium (no statistically significant differences, paired *t*-test). On the other hand, immunostimulation was observed when culturing resting PBMCs with hMSC (Figure 1(c)). The presence of xeno-free hMSC increased 260 ± 153%, the number of proliferative PBMCs, and the control hMSC increased 280 ± 167% (no statistically significant differences, paired *t*-test).

We further analysed the levels of TNF-*α* in these cultures (Figure 1(d)). TNF-*α* levels were low in resting PBMCs (R), independently of their coculture with hMSC and medium used. Mitogen-stimulated PBMCs secreted higher levels of TNF-*α* when compared with resting PBMCs (*p* < 0.05, *n* = 4, Kruskal-Wallis test followed by Dunn's multiple comparison test). In the presence of hMSC, the levels of TNF-*α* were no longer different from the unstimulated control (R). And although it is not statistically significant, there was a tendency of hMSC to decrease the production of TNF-*α* by mitogen-stimulated PBMCs (19 ± 12% reduction in XF-hMSC and 40 ± 29% in FBS-hMSC). Generally, TNF-*α* levels were lower in XF-Medium than those in FBS-Medium.

Taken together, these data indicate that expansion of hMSC in XF-Medium did not interfere with the immunosuppressive and immunostimulatory properties of the cells.

### 3.2. Xeno-Free Expanded hMSC Are Attracted by Macrophages

**Figure 2:**
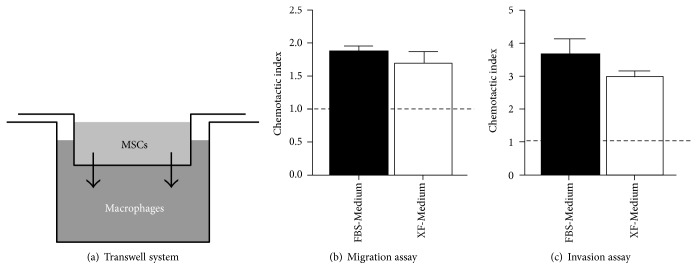
hMSC expanded in XF-Medium maintain their chemotactic properties towards macrophages. Recruitment assays were performed in 24-well plates using a transwell chamber (a). The lower compartment was filled with serum-free medium (negative control) or serum-free medium with macrophages. hMSC expanded in FBS-Medium or XF-Medium were added to the upper compartment and allowed to migrate for 7 hours in the migration assay (b) or for 24 hours in the invasion assay performed with the membrane coated with Matrigel (c). No statistical differences were observed amongst the different media used (*n* = 4, Mann-Whitney test). The dashed line indicates the chemotactic index in the negative control (1).

The capacity of hMSC to be recruited to an inflammation area is important for many of its clinical applications. It has been shown that hMSC can be recruited by immune cells, such as macrophages, NK cells, and T cells [25–27, 22]. To study whether hMSC expanded under xeno-free conditions can be recruited by macrophages, a migration and an invasion assays were performed. These assays are based on the Boyden chamber principle, with hMSC cultured on the top chamber and macrophages on the bottom chamber (Figure 2(a)). The transwell system used for the invasion assay was coated with Matrigel, giving us information about both the migratory capacity and also the capacity of hMSC to degrade and invade through extracellular matrix. This invasion ability of hMSC is important for the cells to reach the place of injury and/or inflammation. In migration assays, membranes are coated with gelatine at a very low concentration, so that adhesion is promoted but a matrix is not formed, giving us a measure of cell migration alone.

**Figure 3:**
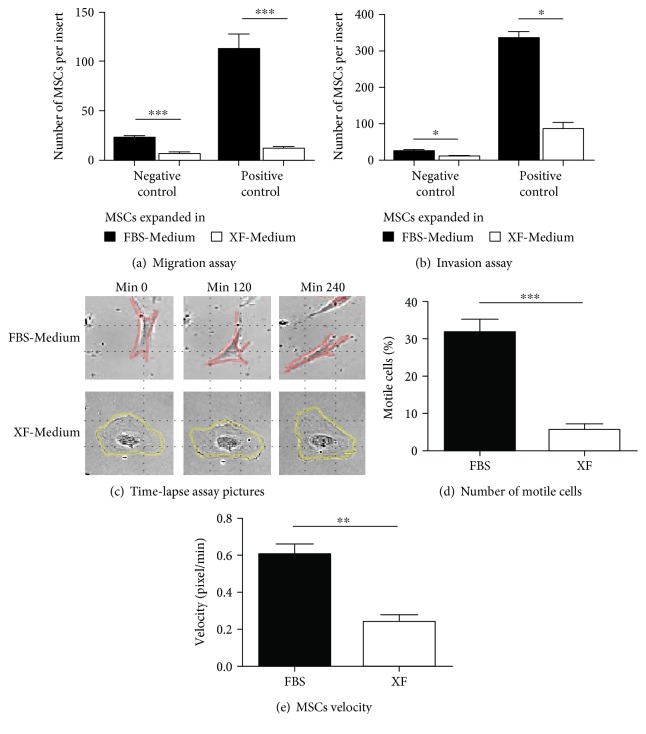
Motility of hMSC cultured in XF-Medium. (a-b) Recruitment assays**—**the lower compartment was filled with serum-free medium (negative control) or with medium supplemented with 30% FBS (positive control); hMSC were expanded in XF-Medium or FBS-Medium (*n* = 4, Mann-Whitney test). (c–e) Time-lapse analysis**—**hMSC expanded in FBS-Medium or XF-Medium were incubated overnight in DMEM without supplements. Snapshots were taken every 10 minutes for 12 hours (c). The percentage of motile cells was calculated counting the number of motile cells and the total number of cells that appear in each video (*n* = 10; unpaired *t*-test) (d). The velocity of 3 randomly selected cells in each video was calculated using Fiji program. The average velocity from hMSC expanded in FBS-Medium and XF-Medium is shown (*n* = 36; Mann-Whitney test) (e).

Macrophages were able to recruit hMSC cultured in XF-Medium in both migration and invasion assays, showing that xeno-free expanded hMSC maintain their chemotactic and matrix remodelling properties (Figures 2(b) and 2(c)). No significant differences were observed between the use of XF-Medium or FBS-Medium to expand hMSC (Man-Whitney test). Although hMSC expanded under xeno-free conditions showed a chemotactic response towards macrophages, the number of cells that crossed the membranes in both the negative control and the experimental condition was reduced (69–90% reduction in the migration assay, and 54–74% reduction in the invasion assay), which was also verified with a positive control (Figures 3(a) and 3(b)). To clarify whether xeno-free hMSC present an impaired motility, we performed time-lapse microscopy analysis by imaging for 12 hours for three different hMSC donors. Quantification of the number of motile cells and the cells velocity showed impairment in the motility of hMSC expanded in XF-Medium (Figures 3(d) and 3(e)). Careful observation of the movies indicated that the front of hMSC expanded in XF-Medium could not adhere to the substrate and thus cells did not move in a certain direction (Figure 3(c)).

**Figure 4:**
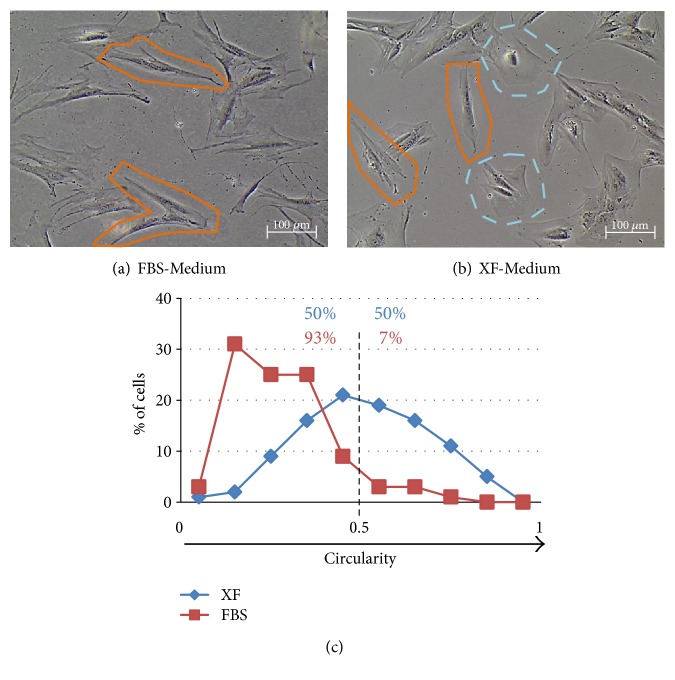
Bright-field images of hMSC cultured in control (FBS-Medium) (a) and XF-Medium (b) in passage 5 after 6 days in culture. Elongated cells with sharp ramifications are highlighted in orange (continuous line), and cells with a more polygonal and rounded shape are highlighted in blue (discontinuous line). (c) Frequency distribution of hMSC circularity of cells expanded in FBS- or XF-Medium (3 samples, 2 fields per sample, 13–20 cells per field counted; *n* = 100).

Careful observation suggested that the morphology of cells cultured in this medium was slightly different from the one observed in FBS-Medium (Figures 4(a) and 4(b)). It was observed that cells in FBS-Medium presented a more elongated shape with sharp ramifications, while cells in XF-Medium were more heterogeneous, including cells with elongated shape but also polygonal and circular cells. This observation was corroborated by morphological quantification: 94% of the hMSC cultured in FBS-Medium presented a circularity factor lower than 0.5, while hMSC cultured in XF-Medium presented a more heterogeneous morphology, only 50% of the cells presenting a circularity factor lower than 0.5 (Figure 4(c)). This change in morphology could be related with differences on cell polarization, which might be linked with differences on hMSC motility. Therefore, xeno-free expanded hMSC maintained their chemotactic and immunoregulatory properties but showed impaired motility in comparison with hMSC expanded in FBS.

## 4. Discussion

There is a need for developing new animal-free media to expand hMSC for use in human therapies. Previous studies have shown that a human plasma-derived supplement for cell culture (SCC) is a potential candidate to substitute FBS. Díez et al. showed that this product can be used to expand hMSC, maintaining their phenotype and multipotentiality [50]. In this present study, we have shown that SCC does not interfere with human hMSC chemotactic and immunomodulatory properties.

Díez and colleagues reported a higher growth rate of hMSC when compared with hMSC expanded in the recommended commercial medium, which was not observed here [50]. Despite of the different proliferation rate, here, it was found that hMSC could be successfully expanded in XF-Medium without platelet lysate. In Díez et al., medium included platelet lysate and basic fibroblast growth factor; the reference medium used was different from the one used here; and hMSC donors were younger [50]. It is important to consider that the age of hMSC donors, especially in therapies using autologous cells, is normally high, as most people in need of these therapies are the elderly.

The immunomodulatory properties of hMSC are of high interest to treat immune-related diseases. Other studies performed in xeno-free conditions only studied hMSC immunosuppression and showed that hMSC expanded in medium-containing platelet lysate have reduced immunosuppressive properties than hMSC expanded in FBS-containing medium or serum-free medium [41, 60, 61]. Here, we studied not only the immunosuppressive hMSC properties but also the immunostimulatory ones. For this, hMSC were cocultured with resting PBMCs, with mitogen-stimulated PBMCs, or with a mixed leukocyte reaction. The results obtained demonstrate that hMSC were able to suppress proliferation of mitogen-stimulated PBMCs independently of their expansion medium (XF- or FBS-Medium). Furthermore, xeno-free expanded hMSC and control MSC were able to stimulate proliferation of resting PBMCs, thus maintaining their immunostimulatory properties. The results obtained in the mixed leukocyte reaction were diverse, with hMSC acting as immunosuppressors or immunostimulators depending on the PBMC response. Our results agree with the current view that the immunomodulatory properties of hMSC depend on the niche; in an inflammatory niche, cytokines prime hMSC, so they can act as immunosuppressor agents, but in a niche where no proinflammatory cytokines are present, hMSC act as immunostimulatory agents [14, 15, 62].

Activation of PBMC proliferation with a mitogen (PHA) led to increased TNF-*α* level. Introducing hMSC into this proinflammatory culture led to a decrease in TNF-*α* level, both with xeno-free and control-expanded hMSC. These results agree with the idea that activated T lymphocytes secrete TNF-*α*, which then prime hMSC to act as immunosuppressors and block TNF-*α* release [63, 64]. TNF-*α* levels did not change when PBMCs were resting, suggesting that TNF-*α* is only related with hMSC immunosuppressive properties but not the immunostimulatory ones. TNF-*α* levels were overall lower when using SCC as supplement than when using FBS, suggesting that XF-Medium might be affecting cytokine production by the PBMC.

hMSC chemotactic response towards cells of the immune system is crucial in therapies where the hMSC are implanted systemically or far from the site of inflammation. Cells involved in the immune response (such as macrophages and NK cells) are able to attract hMSC [25]. Here, xeno-free expanded hMSC maintained their capacity to be attracted by macrophages. We analysed not only the chemotactic response of xeno-free expanded hMSC (migration assay) but also their ability to degrade the matrix, using Matrigel to cover the membrane (invasion assay). MSC showed a higher chemotactic index in the invasion assay than in the migration assay. The chemotactic index is obtained by dividing the number of cells that migrated in the experimental condition by the number of cells that migrated in the negative control. As it is more challenging for the cells to cross the Matrigel on the invasion assay, hardly any cell could cross the membrane in the absence of macrophages. We can speculate that the strong effect seen in the presence of macrophages might be due to the fact that besides producing the chemoattractants that are important to stimulate migration, these cells also produce MMPs that contribute to matrix remodelling. In the case of the migration assay, without any Matrigel, a few more cells can cross the membrane in the negative control; thus, the difference with the number of cells recruited by macrophages is lower, and the resultant chemotactic index is reduced.

Although the chemotactic properties of xeno-free expanded hMSC were preserved, the motility of hMSC was impaired, as found in time-lapse assays. This impairment in cell motility may be related with the morphological changes observed as the most common motility process used by mammalian cells involves polarized actomyosin-driven shape change [65]. It is important that future studies with xeno-free media consider its impact on the cell motility as new formulations should promote not only the cell immunomodulatory properties but also their homing capacity.

It has previously been shown that hMSC grown in SCC-containing medium maintain their hMSC characteristics, including expression of typical hMSC proteins and differentiation potential into adipocytes, chondrocytes, and osteoblasts [50]. In Díez et al. [50], commercial bone marrow-derived hMSC were expanded in the same medium used here but with additional platelet lysate and fibroblast growth factor. Platelet lysate supplementation is controversial, and its effect on immunomodulatory MSC properties is not clear, with some studies showing that it reduces hMSC immunosuppressive properties [40, 41, 61, 66]. Therefore, in this work, we tested the effect of SCC-based medium without platelet lysate. The addition of platelet lysate, together with fibroblast growth factor, may have led to a higher proliferation rate in the previous study. Also, this proliferation may have been affected by the age of the MSC donors. Here, we opted to use cells from older donors, as it is likely that most people in need of therapies using autologous cells are the elderly. Notwithstanding, and despite these differences, hMSC in XF-Medium without platelet lysate could be successfully expanded in vitro, while maintaining their immunomodulatory properties.

Apart from preserving hMSC immunomodulatory and chemotactic properties, SCC-based xeno-free medium presents some advantages compared with other available xeno-free media. SCC is a GMP product that undergoes different safety steps to avoid the transmission of any disease and that is produced from more than 10,000 donations, reducing the variability between lots, while being. Platelet lysate would also be an interesting xeno-free supplement, but high variability is expected if the number of donors is low and it might impact on immunomodulation. As Bobis-Wozowicz et al. showed, different media can have different effects on each hMSC; thus, it is highly recommended to test which is best for each particular application [45].

## 5. Conclusion

Here, we report expansion of hMSC in a xeno-free medium without platelet lysate while preserving the cell immunomodulatory and chemotactic properties. As SCC is an industrial GMP product from human origin obtained from a large number of healthy donors, and with an improved safety margin, SCC can be a potential candidate to culture hMSC for human cell therapies.

## Supplementary Material

Supplementary Figure 1. Schematic representation of an Immunosuppression Assay. CFSE-Labelled Responder Lymphocytes are co-cultured with Mitomycin-inhibited Stimulator Cells from un-related donors. In a MLR, Responder Cells recognize the allogenic Stimulator Cells, secreting cytokines and stimulating their proliferation. When Responder Cells and Stimulator Cells are co-cultured together with hMSC, the cytokines produced by the Responder Cells prime hMSC. Primed-hMSC act as immunosuppressors, inhibiting Responder's proliferation.

## Abbreviations

DMSO: Dimethyl sulfoxide
FBS: Foetal bovine serum
GvHd: Graft-versus-host-disease
hMSC: Human mesenchymal stem/stromal cells
IDO: Indoleamine 2,3-dioxygenase
IFN-*γ*: Interferon gamma
MLR: Mixed leukocyte reactions
PBMC: Peripheral blood mononuclear cells
PFA: Paraformaldehyde
PGE2: Prostaglandin E2
PHA: Phytohemagglutinin
PRC: Proliferative responder cells
SCC: Supplement for cell culture
TNF-*α*: Tumor necrosis factor alpha
XF: Xeno-free.
