# Supplementary material for: Human Bone Marrow Mesenchymal Stem/Stromal Cells Preserve Their Immunomodulatory and Chemotactic Properties When Expanded in a Human Plasma Derived Xeno-Free Medium
Source: Stem Cells Int. 2017 May 14;2017:2185351. doi: 10.1155/2017/2185351 (PMC5446864; doi:10.1155/2017/2185351)
Supplement: Supplementary file 1 — Supplementary Figure 1. Schematic representation of an Immunosuppression Assay. CFSE-Labelled Responder Lymphocytes are co-cultured with Mitomycin-inhibited Stimulator Cells from un-related donors. In a MLR, Responder Cells recognize the allogenic Stimulator Cells, secreting cytokines and stimulating their proliferation. When Responder Cells and Stimulator Cells are co-cultured together with hMSC, the cytokines produced by the Responder Cells prime hMSC. Primed-hMSC act as immunosuppressors, inhibiting Responder's proliferation. [file 2185351.f1.pptx]

## Slide 1
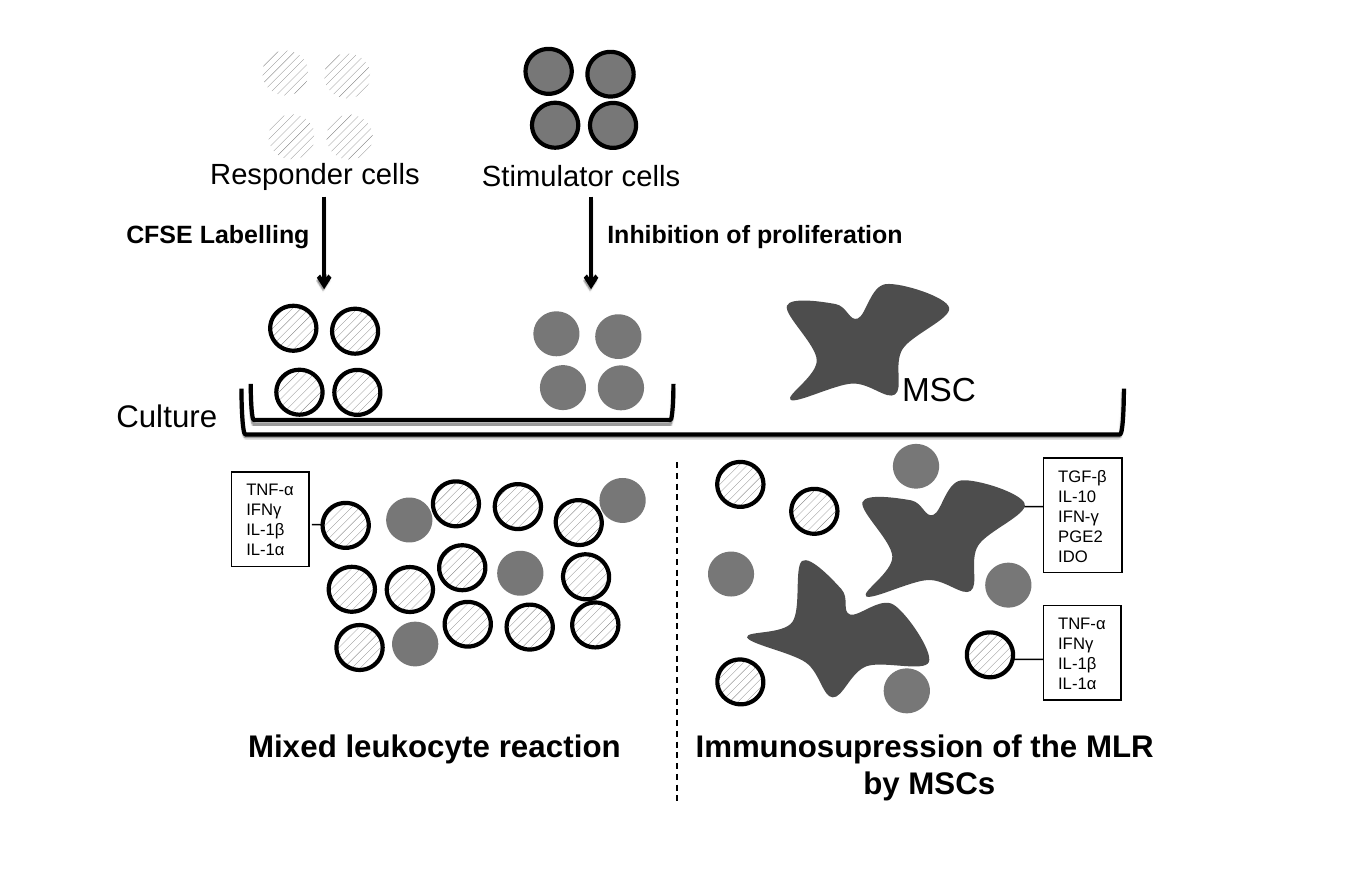

Responder cells
Stimulator cells
CFSE Labelling
Inhibition of proliferation
MSC
Culture
TGF-β
IL-10
IFN-γ
PGE2
IDO
TNF-α
IFNγ
IL-1β
IL-1α
TNF-α
IFNγ
IL-1β
IL-1α
Mixed leukocyte reaction
Immunosupression of the MLR
by MSCs
